# Miller Fischer syndrome after COVID-19 infection and vaccine: a systematic review

**DOI:** 10.1007/s13760-023-02336-5

**Published:** 2023-07-19

**Authors:** Panayiota Neophytou, Artemios Artemiadis, Georgios M. Hadjigeorgiou, Panagiotis Zis

**Affiliations:** 1 Medical School, University of Cyprus, Old Road Nicosia-Limmasol 215/6, 2029 Nicosia, Cyprus; 2 Medical School, National Kapodistrian University of Athens, 15772 Athens, Greece

**Keywords:** COVID-19, Infection, Vaccine, Miller Fisher, Syndrome, GQ1b, Post-infectious

## Abstract

**Background:**

COVID-19 (CoranaVirus disease 2019) is an ongoing infectious disease caused by the RNA SARS-CoV-2 virus (Severe Acute Respiratory Syndrome CoronaVirus-2). The virus mainly causes respiratory symptoms, but neurological symptoms have also been reported to be part of the clinical manifestations of the disease. The aim of this study was to systematically review Miller fisher syndrome (MFS) published cases, in the context of COVID-19 infection or vaccination.

**Methods:**

A systematic literature review on Medline was performed. A total of 21 papers were included in the present review.

**Results:**

Twenty-two MFS cases (77% males) were identified, 14 related to COVID-19 infection and 8 to vaccination against COVID-19. The median age of the adult patients was 50 years (interquartile range 36–63 years). Sixteen patients (73%) had the classic triad of MFS (ophthalmoplegia, ataxia, areflexia), four (18%) had acute ophthalmoplegia and one other characteristic symptom and two patients (9%) had only one other characteristic symptom, but they tested positive for GQ1b antibodies. Nine (41%) patients had positive GQ1b antibodies and were classified as “definite” MFS. Albuminocytologic dissociation was found in half of the cases. The outcome was favourable in the majority of cases (86%) whereas one patient, despite the initial improvement, died because of a cardiac arrest, after cardiac arrythmia.

**Conclusions:**

MFS after COVID-19 infection/vaccination was found to have the typical epidemiological characteristics of classic MFS; being rare, occurring more often after infection than vaccination, affecting mainly middle-aged males usually within 3 weeks after the event and having an excellent prognosis after treatment with IVIG or even with no treatment at all. We found no evidence that MFS after COVID-19 infection was different from MFS after COVID-19 vaccination, although the former tended to occur earlier.

**Supplementary Information:**

The online version contains supplementary material available at 10.1007/s13760-023-02336-5.

## Introduction

COVID-19 (CoronaVirus disease 2019) is an ongoing global health emergency caused by the RNA SARS-CoV-2 virus (Severe Acute Respiratory Syndrome CoronaVirus-2). It was declared by WHO (World Health Organization) as a world pandemic on 11th of March, 2020 [1]. The virus mainly causes respiratory symptoms, but neurological symptoms have also been reported to be part of the clinical manifestations of the disease. A review that included 841 patients hospitalized in Europe with COVID-19 showed that 57.4% of them developed some form of neurological symptoms [2].

Current literature suggests that neurological symptoms and manifestations resulting from COVID-19 can occur prior, during, or after respiratory involvement. The most common neurological symptoms associated with COVID-19 are fatigue, myalgia, headache, impaired consciousness, dizziness, ageusia, and anosmia; and less often reported symptoms include visual impairment, neuropathic pain, occipital neuralgia, ataxia, tremor, and tics [3]. Growing number of case reports and/or series indicate that a variety of neurological conditions and post-viral triggered autoimmune complications, occur in association with SARS-CoV-2 infection, which mainly include Guillain-Barré syndrome (GBS), myopathy, encephalopathy, meningoencephalitis, encephalomyelitis, and myelitis. Suggested mechanisms are direct SARS-CoV-2 infection to the nervous system, neuroinflammation, post-viral triggered autoimmune response, hypercoagulability, and metabolic or hypoxic injury [3, 4].

As GBS is an infection-related demyelinating polyradiculoneuropathy it was only a matter of time before an association with SARS COV2 virus was reported. Some reviews of multiple cases of GBS associated with COVID-19 have been published [5, 6]. In most cases, the onset of the neurological symptoms related to GBS was during infection (i.e., parainfectious) or about 1 to 4 weeks after the diagnosis of COVID-19 (post-infectious) [7].

In this paper we will focus on COVID-19-related Miller fisher syndrome (MFS), a variant of GBS characterized by acute external ophthalmoplegia, areflexia or ataxia and positive GQ1b antibodies in the serum that has not been systematically reviewed in the context of COVID-19 infection or vaccination so far. The main aim of this review is to describe the clinical characteristics including outcome of published cases in this context.

## Methods

This study was reported in accordance with the Preferred Reporting Items for Systematic Reviews and Meta-Analysis (PRISMA) guidelines.

### Selection processes and eligibility criteria

A systematic literature search was performed on the 14th of October 2022, in the Medline database to identify reports of COVID-19 and/or COVID-19 vaccinated patients presenting with MFS. Two medical subject heading (MeSH) terms were used; Term A was ‘COVID OR SARS-CoV-2’ and Term B was ‘Miller Fisher’. No filters were applied. The reference lists of articles that met the eligibility criteria were further screened to identify additional studies that may fall within the scope of this review.

COVID-19 infection was documented with either rapid antigen or PCR test for SARS-COV-2. MFS syndrome was defined as “definite MFS” if positive GQ1b antibodies were present with any of the three characteristic clinical manifestations and “probable MFS” if antiGQ1b were Negative or not mentioned but there were clinical characteristics highly suggestive of MFS.

All articles eligible to be included in this review met the following inclusion criteria: (1) human subjects were involved, (2) the full article was retrieved in English language and (3) articles reported cases of MFS following COVID-19 infection or vaccination. Articles meeting the following criteria were excluded from our review: (1) non-original articles (i.e., review articles, guidelines etc.), (2) studies that didn’t sufficiently document recent COVID-19 infection or vaccination and (3) articles presenting cases not fulfilling MFS criteria.

### Data collection process

Data were extracted from each study in a structured coding scheme using Excel. Data collected included clinical characteristics (presence of ophthalmoplegia, areflexia or ataxia), demographics, past medical history, means of COVID-19 diagnosis OR what vaccine was administered, symptoms, time of onset. Of neurological symptoms from infection (based on clearly documented timeline of symptoms suggestive of COVID-19 as for example cough, fever, dyspnoea) or vaccination, antibodies tested, cerebrospinal fluid analysis (white blood cell count and protein), treatment and outcomes. When there was uncertainty regarding the data, at least two authors discussed the study in question to ensure consensus.

### Study risk of bias assessment

The quality of included case reports was assessed by a modified version of the Joanna Briggs Institute Critical Appraisal Checklist for Case Reports, by using seven out of the eight yes/no/unclear questions (1 item was deemed not applicable for this study)[8]. To summarize the overall quality of case reports, these were grouped into the following categories: “High risk of bias” (studies that met up to 2 of the quality criteria), “Moderate risk of bias (studies that met 3–5 of the quality criteria)” and “Low risk of bias” (studies that met 6 or 7 of the quality criteria). The quality assessment is available as Supplementary material.

### Data synthesis

In our case tabulating collected data is the main method of data synthesis and presentation.

## Results

### Study selection

**Fig. 1:**
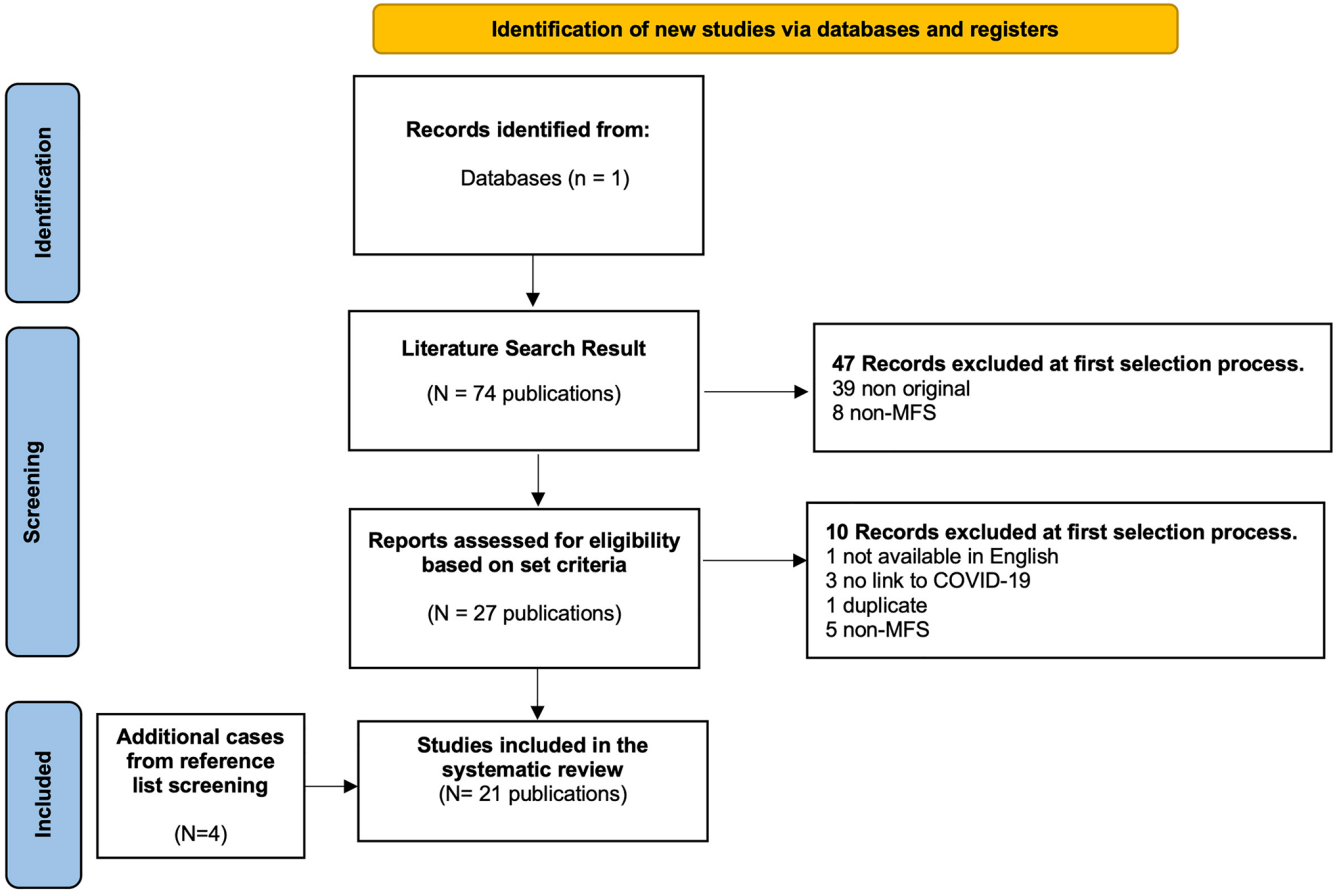
PRISMA chart displaying the screening and study selection process

Our search strategy produced a total of 74 results. During the eligibility assessment 57 articles were excluded. Four articles were added after perusing the reference lists of the included papers. Ultimately, a total of 21 completed reports, reporting 22 MFS patients related to COVID-19 infection (n = 14) or vaccination (n = 8) were available and were included in the present review. [9–29] These were published between 2020 and 2022. The selection process is illustrated in Fig. 1 (PRISMA chart).

### Demographics and co-morbidities

Tables 1 and 2 summarize the characteristics of the included cases. There were 14 patients in the COVID-19 infection group and 8 in the vaccination group. Five patients had received the BNT162b2 mRNA COVID-19 vaccine (Pfizer^®^), two had received the ChAdOx1-SARS-COV-2 recombinant vaccine (Oxford/AstraZeneca^®^) and one the Sinovac-CoronaVac vaccine.

**Table 1:** Summary of characteristics of COVID-19-related Miller-Fisher syndrome cases

Reference	Gender	Age (years)	History	Vaccination	Ophthalmoplegia	Ataxia	Areflexia	Sensory symptoms	Weakness	Other cranial nerve	Dysautonomia	Onset of COVID-19 symptoms, days	Serology	Miller Fisher diagnosis	CSF	Treatment	Outcome
Kopscik et al. [9]	M	31	Clear	N/A	Υes	Υes	Υes	Υes	Υes	Facial n., Hypoglossal n	No	Diagnosed during admission	Anti-GQ1b	Definite	Normal	IVIG + PLEX	Poor
Kajani et al. [10]	M	50	Diabetes	N/A	Υes	Υes	Υes	No	Υes	Vagus n	No	Diagnosed during admission	Negative	Probable	Normal	IVIG	Death
Ray et al. [11]	M	63	Hypertension	N/A	Υes	Υes	Υes	Υes	No	–	No	1	Not tested	Probable	High protein	Nil	Improvement
Tran et al. [12]	M	42	Diabetes	N/A	Υes	Υes	No	No	Υes	Facial n	No	Diagnosed during admission	Not tested	Probable	No cells, Increased protein (> 100 mg/dL)	IVIG	Improvement
Lowery et al. [13]	M	45	Dyslipidemia, hypertension, Crohn’s disease	N/A	Υes	Υes	Υes	Υes	Υes	–	No	14	Anti-GQ1b	Definite	Normal	IVIG	Poor
Senel et al. [14]	M	61	Nothing reported	N/A	Υes	Υes	Υes	Υes	No	–	No	17	Negative	Probable	No cells, Increased protein (> 100 mg/dL)	IVIG	Improvement
Aljomah et al. [15]	M	9	Clear	N/A	Υes	Υes	Υes	Υes	Υes	–	No	Diagnosed during admission	Anti-GQ1b	Definite	NR	IVIG	Improvement
Dinkin et al. [16]	M	36	Clear	N/A	Υes	Υes	Υes	Υes	No	–	No	4	Negative	Probable	NR	IVIG	Improvement
Lantos et al. [17]	M	36	Clear	N/A	Υes	Υes	Υes	Υes	No	–	No	2	GM1 (equivocal)	Probable	NR	IVIG	Improvement
Gutiérrez-Ortiz et al. [18]	M	50	Asthma	N/A	Υes	Υes	Υes	Υes	No	–	No	3	Anti-GD1b	Probable	No cells, Increased protein (< 100 mg/dL)	IVIG	Improvement
Gutiérrez-Ortiz et al. [18]	M	39	Clear	N/A	Υes	No	Υes	No	No	–	No	3	Not tested	Probable	No cells, Increased protein (< 100 mg/dL)	Nil	Improvement
Biswas et al. [19]	F	55	Clear	N/A	No	No	Υes	Υes	No	–	Yes	9	Anti-GQ1b, Anti-GD1b	Definite	No cells, Increased protein (> 100 mg/dL)	IVIG	Improvement
Manganotti et al. [20]	F	50	Clear	N/A	Υes	Υes	Υes	Υes	No	–	No	16	Not tested	Probable	No cells, Increased protein (< 100 mg/dL)	IVIG	Improvement
Costa et al. [21]	F	21	Nothing reported	N/A	Υes	Υes	Υes	No	No	–	No	21	Anti-GQ1b	Definite	NR	IVIG	Improvement

**Table 2:** Summary of characteristics of vaccine against COVID-19 -related Miller-Fisher syndrome cases

References	Gender	Age (years)	History	Vaccination	Ophthalmoplegia	Ataxia	Areflexia	Sensory symptoms	Weakness	Other cranial nerve	Dysautonomia	Onset since vaccine, days	Serology	Miller Fisher diagnosis	CSF	Treatment	Outcome
Dang et al. [22]	M	63	Clear	ChAdOx1-SARS-COV-2	Υes	Υes	Yes	Υes	Υes	Facial	No	14	Negative	Probable	No cells, Increased protein (> 100 mg/dL)	IVIG	Improvement
Nishiguchi et al. [23]	M	71	Diabetes	BNT162b2 (1st dose)	Υes	Υes	No	No	No	–	No	18	Negative	Probable	No cells, Increased protein (< 100 mg/dL)	IVIG	Improvement
Yamakawa et al. [24]	M	30	Nothing reported	BNT162b2 (2nd dose)	Υes	Υes	Υes	No	No	–	No	7	Anti-GQ1b and anti-GT1a immunoglobulin G (IgG)	Definite	Normal	IVIG	Improvement
Michaelson et al. [25]	M	78	Clear	BNT162b2 (2nd dose)	Υes	Υes	Υes	Υes	No	–	No	13	Anti-GQ1b (equivocal)	Definite	No cells, Increased protein (> 100 mg/dL)	IVIG	Improvement
Abičić et al. [26]	F	28	Clear	BNT162b2 (1st dose)	Υes	No	No	No	No	–	No	18	Anti-GQ1b	Definite	Normal	IVIG	Improvement
Kim et al. [31]	F	84	Nothing reported	ChAdOx1-SARS-COV-2	Υes	Υes	Υes	No	No	–	No	8	Anti-GQ1b	Definite	NR	No	Improvement
Sansen et al. [29]	M	65	Diabetes	BNT162b2 (1st dose)	Υes	Υes	No	No	No	–	No	35	Anti-sulfatide	Probable	No cells, Increased protein (> 100 mg/dL)	IVIG	Improvement
Siddiqi et al. [28]	M	53	Hypertension	Sinovac	Υes	Υes	Υes	Υes	Υes	–	No	8	Not tested	Probable	No cells, Increased protein (> 100 mg/dL)	No	Improvement

Of the 22 patients 17 were males (78.6% within the COVID-19 group and 75% in the COVID-19 vaccine group) and 5 females. One male patient within the COVID-19 infection group was 9 years old. The remaining cases were adults with a median age of 50 years (interquartile range 36–63 years).

Within the COVID-19 infection group the adult patients had a median age of 45 years (interquartile range 36–53 years) and within the COVID-19 vaccine group there were patients with a median age of 41 years (interquartile range 36–76 years). The difference was not statistically significant (Mann–Whitney U Z = − 1.95, p = 0.051), not even after including the 9-year-old case (Mann–Whitney U Z = − 1.78, p = 0.075), or only considering definite MFS cases (data not shown). Four patients had diabetes mellitus (two in the vaccine group) and one patient had a history of Crohn’s disease and was immunosuppressed.

### Miller fisher syndrome presentation and diagnosis

Τwenty-one (95%) patients suffered from acute ophthalmoplegia, 18 (82%) from ataxia and 18 (82%) from areflexia. Sixteen patients (73%) had the classic triad of MFS, four (18%) had acute ophthalmoplegia and one other characteristic symptom and two patients (9%) had only one other characteristic symptom, but they tested positive for GQ1b antibodies. Four patients had also one other cranial nerve involved, mainly facial nerve and one patient presented dysautonomia. Nine (41%) patients had positive GQ1b antibodies and were classified as “definite” MFS; 5 (35.7%) within the COVID-19 infection group and 4 (50%) within the COVID-19 vaccine group. One patient was positive for anti-GD1b antibodies, and one was equivocally positive for GM1 antibodies. Also, one patient in the vaccine group was double positive for GQ1b and GT1a antibodies. Albuminocytologic dissociation was found in half of the cases.

### Time since COVID-19 infection or vaccination

All reports of vaccine-related MFS documented the time of neurological symptoms since vaccination. Four reports of infection-related MFS documented that COVID-19 was diagnosed on admission without providing a clear timeline of any COVID-19 symptoms and, therefore, were not considered in analysis.

The median time from COVID-19 infection or vaccination to MFS was 11 days (interquartile range 4–17 days). The median from COVID-19 infection to MFS was 7 days (interquartile range 3–16 days). The median from COVID-19 vaccination to MFS was 14 days (interquartile range 8–18 days). The between group difference was found to be significant (Mann–Whitney U test, Z = − 2.17, sig. 0.030).

### Treatment and outcome

In the infection group, twelve patients (86%) received treatment with IVIG (one patient received IVIG and plasma exchange) whereas two patients did not receive treatment and improved spontaneously. Of the treated patients, two showed minimal or no improvement and one, despite the initial improvement, died because of a cardiac arrest, after cardiac arrythmia. In this group, two of the treated patients did not have any signs or symptoms on follow up evaluation, one only had residual anosmia, ageusia and two others only presented with generalized hyporeflexia. Some more profound residual symptoms in other patients include ataxia, diplopia and fatigue.

In the COVID-19 vaccine group, six patients (75%) received intravenous immunoglobulins and clinically improved, with two of them being free of symptoms on follow up evaluation. Two patients spontaneously improved without treatment. Residual symptoms mentioned were bilateral facial and bilateral proximal lower limb weakness, some dysmetria and paresthesiae, and double vision only in the far right and left eye position.

## Discussion

This study aimed to systematically describe the clinical characteristics of MFS after COVID-19 infection or vaccination. Firstly, there were more cases in the literature of MFS after COVID-19 infection than vaccination, though this observation may be due to publication bias and therefore should be interpreted with caution. Overall, we found that all ages and genders could be affected, although mostly middle-aged males were reported. There was no age- or gender-specific difference between the two COVID-19 groups explored, albeit the sample was too small due either the rare incidence of MFS or MFS after COVID-19 infection/vaccination or both. In this limited number of patients, MFS occurred immediately after the COVID-19 infection/vaccination up to about 5 weeks after. We found a predilection of an earlier presentation of about one week in the COVID-19 infection group compared to the vaccination group, and this difference was found to be statistically significant. However, this finding should be interpreted with caution as often COVID-19 is asymptomatic and, therefore, the exact period between MFS and COVID018 is prone to recall bias. Contrary, the date of vaccination is always documented and not prone to recall bias. Most of the treating physicians preferred to use IVIG for managing these cases. With only three exceptions in the COVID-19 infection group, most patients showed an excellent outcome with few or no residual symptoms. Our results are in keeping with earlier reviews that included smaller number of patients [30, 31].

The clinical hallmark of MFS is a triad presentation of acute ophthalmoparesis, areflexia, and ataxia. The triad was first described as a variant of the GBS in 1932 by Collier. In 1956, Miller Fisher classified it as a unique entity within the GBS spectrum. The incidence of MFS is approximately 1–2 per 1,000,000 persons, with men affected more often than women, as found in our study. Cranial nerve involvement is typical, resulting in facial, oculomotor, or bulbar weakness, which may extend to the limbs. The neurologic symptoms in MFS usually start within 8–10 days after the illness or vaccination, which is similar to the cases described in this review [32].

Miller fisher syndrome is highly associated with the presence of antibodies to GQ1b ganglioside (anti-GQ1b) in the acute phase, but the absence of antibodies does not rule out the disease completely. Anti GQ1b antibodies are found positive in 70–90% of Miller fisher syndrome patients [33]. This finding in conjunction with the absence of anti-GQ1b antibodies in healthy controls and patients with other diseases indicate this test’s high specificity for the disease. In our review anti GQ1b antibodies were found positive in 45% of the patients, which is lower than expected but conclusions are difficult to be made due to the small patient sample included.

Albuminocytologic dissociation is present when analyzing the CSF in 90% of patients at peak disease, and in only half of them on initial analysis, like in this study. Approximately 10% of patients with GBS have normal CSF studies. Approximately 15–20% have a mild increase in cerebrospinal fluid cell count (5–50 cells/microliter) [34].

Intravenous immunoglobulin (IVIg) and plasma exchange (PLEX) are the mainstay of MFS treatment, although some patients may not require immunotherapy. The prognosis of Miller fisher syndrome is usually good with a case fatality of less than 5%. The mean recovery times range between 8 and 12 weeks [35]. Residual symptoms were present in a minority of cases, and a reoccurrence of the Miller fisher syndrome was reported in some patients. Death is reported in some cases but most of the time a co-morbitity exists [36]. These are in accordance with the cases described in this review.

Referring to the etiology of MFS, the ganglioside GQ1b is richly present at the paranodal myelin in cranial nerves III, IV and VI and in the dorsal root ganglia and muscle spindles. This localization is responsible for the unique clinical triad of Miller Fisher’s. The trigger for the development of antiganglioside antibodies appears to frequently be an infection most likely through an immune mechanism of “molecular mimicry” between foreign and innate antigens [37]. We suppose that a similar mechanism takes place after COVID-19 infection or vaccination.

This study suffers from several limitations. Firstly, only 41% of the reported patients were GQ1b antibody positive and were classified as definite MFS cases. However, most patients had other clinical characteristics of MFS thus the risk of misdiagnosis was assumed to be low. Secondly, five of the 21 reports were found with moderate to high risk of bias thus, our findings should be delt with caution. Most authors failed to sufficiently describe the history timeline and clinical follow-up. Finally, there is always the chance that we have missed pertinent reports in the grey literature or in other than Medline databases.

In conclusion and after considering these caveats, MFS after COVID-19 infection/vaccination was found to have the typical well-known epidemiological characteristics of classic MFS; being rare, occurring more often after infection than vaccination, affecting mainly middle-aged males within 3 weeks after the event and having an excellent prognosis after treatment with IVIG or even with no treatment at all. Finally, we found no evidence that MFS after COVID-19 infection was different from MFS after COVID-19 vaccination, although the former tended to occur earlier.

### Supplementary Information

Below is the link to the electronic supplementary material.

Supplementary file1 (XLSX 10 KB)

## Data Availability

The data that support the findings of this study are available from the corresponding author upon reasonable request.
